# Identification of immune signatures predictive of clinical protection from malaria

**DOI:** 10.1371/journal.pcbi.1005812

**Published:** 2017-10-24

**Authors:** John Joseph Valletta, Mario Recker

**Affiliations:** Centre for Mathematics and the Environment, University of Exeter, Penryn Campus, Penryn, United Kingdom; UNSW Australia, AUSTRALIA

## Abstract

Antibodies are thought to play an essential role in naturally acquired immunity to malaria. Prospective cohort studies have frequently shown how continuous exposure to the malaria parasite *Plasmodium falciparum* cause an accumulation of specific responses against various antigens that correlate with a decreased risk of clinical malaria episodes. However, small effect sizes and the often polymorphic nature of immunogenic parasite proteins make the robust identification of the true targets of protective immunity ambiguous. Furthermore, the degree of individual-level protection conferred by elevated responses to these antigens has not yet been explored. Here we applied a machine learning approach to identify immune signatures predictive of individual-level protection against clinical disease. We find that commonly assumed immune correlates are poor predictors of clinical protection in children. On the other hand, antibody profiles predictive of an individual’s malaria protective status can be found in data comprising responses to a large set of diverse parasite proteins. We show that this pattern emerges only after years of continuous exposure to the malaria parasite, whereas susceptibility to clinical episodes in young hosts (< 10 years) cannot be ascertained by measured antibody responses alone.

## Author summary

Understanding naturally acquired immunity against *P. falciparum* malaria is of fundamental importance for malaria control and elimination efforts. The identification of parasite antigens that could potentially be considered as vaccine targets often relies on prospective cohort studies where observed infection rates are related to measured immune responses. However, what is unknown, is how these population-level associations between antibody titres and protection from severe malaria can predict the risk of an infection for an individual. We therefore analysed three sets of cohort-based immune profiles using a machine learning approach in order to identify distinct immune signatures that are predictive of protection at the individual-level. Our results show that even statistically significantly associated responses fail to provide robust information about an individual’s risk of malaria and that machine learning approaches should be considered more prominently alongside traditional methods for analysing these complex and high dimensional datasets.

## Introduction

Naturally acquired immunity to malaria is a complex and poorly understood process, by which individuals living in *P. falciparum* endemic areas develop protection against clinical and symptomatic infections over years of repeated exposure. Since the first experimental evidence demonstrating how passively transferred immunoglobulins from immune adults can dramatically reduce parasitaemia in infected recipients [1, 2] there has been a growing body of evidence that antibody (Ab) responses play an important role for parasite control and protective immunity. However, the unambiguous identification of the target antigens involved has been difficult, and even after decades of research there is still no strong consensus about which candidates could be considered as potential components of an anti-asexual stage vaccine.

Prospective cohort studies, in which individuals’ immune responses against panels of *P. falciparum*-specific antigens at time zero are related to their subsequent risk of developing clinical malaria, have frequently shown how responses to various antigens correlate with increased protection against clinical malaria in an age- and/or exposure dependent manner [3–14]. Proteins expressed by the merozoite life-stage of *P. falciparum*, such as the merozoite surface protein (MSP) or apical membrane protein (AMA), are often the focus of such studies, partially due to their higher sequence conservation compared to other immunogenic but highly polymorphic variant surface proteins (e.g. PfEMP1) that are expressed during the intra-erythrocytic life-stages of the parasite. The protective potentials of anti-merozoite antibodies have been confirmed in *in vitro* and animal studies, which led to those antigens now being considered as potential vaccine targets (see e.g. [15] for a review). However, their contribution to clinical immunity in a field-setting is yet to be quantified.

Small effect sizes and the difficulty in reliably quantifying previous exposure [16] makes the distinction between markers of exposure and markers of protective immunity problematic and has resulted in inconsistent and contradictory findings in the past [17]. More importantly, though, routine analytical approaches based on comparisons between population-level mean responses often fail to convey information about the robustness of the derived associations and how sensitive they are to even small changes in the observed data. The shortcomings of traditional statistical methods are highlighted when trying to predict individual-level protection from population-wide associations. In particular, when dealing with high dimensional data, where a vast number of combinations and interactions must be tested. Here, practitioners typically rely on univariate tests, whilst adjusting for common markers of exposure, thus ignoring potential interplay between different antigens.

Conversely, predictive modelling frameworks based on machine learning offer a systematic way to consider all possible combinations of immune responses against various antigens. These hypothesis-free approaches do not assume *a priori* functional relationships between the measured variables (e.g. Ab-levels) and the response (e.g. the risk of a clinical episode), and test whether these associations could be due to chance (i.e. the ubiquitous *P*-value). Instead, the outcome of interest is the predictive accuracy, i.e. the degree by which the model can predict the response at the level of the individual. They further provide a better understanding of the contribution of individual predictors towards model performance. Thus, machine learning techniques have become popular choices for the analysis of high dimensional datasets in biology and ecology (see e.g. [18–23]).

Here we used a random forests machine learning approach to analyse antibody profiles against panels of *P. falciparum*-specific antigens with the aim to identify signatures that are predictive of an individual’s protective status against clinical malaria episodes. Our results show that immune signatures that clearly distinguish clinically immune individuals can be found only when considering a broad set of antigens from individuals spanning a sufficiently wide age-range, whereas the responses taken from young cohorts are less likely to be informative of the individual’s susceptibility to malaria.

## Materials and methods

### Data

We analysed previously published data from three prospective cohort studies conducted in Kenya, Kenya/Tanzania and Mali, which we simply refer to here as KEN, KTZ and MAL, respectively. The datasets can be found as supporting information (S1 Data, S2 Data and S3 Data). The underlying studies are described in detail elsewhere [8, 12, 24] and summarised in Table 1, so here we just provide a brief overview.

**Table 1:** Overview of the three data sets analysed.

Study site	N	Mean age (years) [range]	No. of antigens	Exposure proxies	Assay
Kenya	121	6.5 [1-10]	36	age, schizont extract	ELISA
Kenya / Tanzania	447	0.95 [0.4-1.5]	46	age, bednets	luminex
Mali	186	8.8 [2-25]	2320*	age, parasite status at screening	microarray

*representing 1204 unique proteins

The KEN dataset contains immune profiles for 286 individuals. However, in line with the original study [12], our analysis was performed on the subset of children who were parasite-positive at screening (N = 121, age = 1-10 years). Immune profiles are ELISA-based antibody titres against 36 *P. falciparum*-specific antigens, taken at the start of the transmission season, with host age and schizont extract reactivity used as exposure proxies. The response variable was incidence of a clinical malaria episode, defined as an axillary temperature of > 37.5°C, plus any parasitaemia for children less than 1 year, and an axillary temperature of > 37.5°C, plus parasitaemia > 2500/*μl* for individuals older than 1 year, during a 6-months follow-up.

The KTZ dataset is based on luminex-derived IgG levels against 46 individual PfEMP1 domains of 447 children (5-18 months old, mean = 11.4 months) living in Kilifi (Kenya) and Korogwe (Tanzania), taken from the placebo arm of the RTS,S malaria vaccine trial [25]. Individuals were followed for an average of 8 months with multiple samples taken over the time course, resulting in a total of 1269 immune profiles. The outcome of interest was the incidence of at least one clinical malaria episode, defined as an axillary temperature of > 37.5°C plus parasitaemia > 2500/*μl*. Age and bednet use were used as proxy variables for exposure risk.

The MAL dataset comprises protein microarray-based antibody reactivity of 186 individuals aged 2-25 years against a panel of 2320 *P. falciparum*-specific epitopes of the 3D7 line, representing 1204 unique proteins (∼ 23% of the *P. falciparum* proteome), taken before the start of the transmission season. The response variable was incidence of clinical malaria, defined as axillary temperature of > 37.5°C plus parasitaemia > 5000/*μl*, over an 8-months period of follow-up. Age and infection status (parasite positive or negative) at first screening were used as exposure proxy variables.

### Random forest predictive modelling

To identify predictive immune signatures underlying clinical protection we employed a random forests [26] machine learning approach, using the *randomForest* package [27] in R [28]. Each dataset (KEN, KTZ, MAL) was analysed separately. As input variables for our models we used the measured immune profiles and, where applicable, their respective exposure proxies. The response variable, i.e. the outcome to be predicted, was the incidence of clinical infections during follow-up as defined by the respective studies. For this work we classified individuals as *susceptible* if they had a recorded episode of clinical malaria within the specified time window, and *protected* otherwise. Unavoidably, protected individuals also included those who may have been uninfected. However, this scenario is probably minimised by the fact that these studies were conducted in villages of moderate to high transmission.

### Feature selection

Random forests are able to deal with data sets where the number of predictors is larger than the sample size. However, when the number of features greatly outweighs the number of samples, such as in the MAL dataset (sample size *N* = 186, number of features *M* = 2320), feature selection is advised to remove uninformative variables and focus on the ones that exhibit sufficient predictive power [29, 30]. We start by first ignoring strongly linearly correlated responses (above a Pearson correlation coefficient of *ρ* = 0.8; which corresponds to around 36% of the original feature set), to avoid biasing the variable importance measures computed by the random forests [31]. Recall that only around half of the measured responses represent unique proteins (see Table 1). Note that these correlated variables are reintroduced in the interpretation stage if any of the features they are associated with have been selected for the final model. The remaining covariates undergo a rigorous supervised feature selection process, based on the mProbes [32] and xRF [33] algorithms, as follows:

(i) fit a large random forests considering all features; keep only the top 30% ranked features according to their variable importance measure for the subsequent steps (this step is justified because most importance scores are very low and it is therefore highly unlikely that any of these features will have sufficient predictive capacity)
(ii) permute the values of every predictor (i.e. antibody response), *X*_*i*_ (*i* = 1 … *M*, where *M* is the total number of predictors), and add these to the original feature space, *S*_*X*_, to generate an extended feature space *S*_*X*,*P*_ (*P* represent the permuted features), which now has the dimension *N* × 2*M* (where *N* is the total number of individuals)
(iii) build a random forests model from {*S*_*X*,*P*_, *Y*}, where *Y* is the response variable (i.e. protected/susceptible)
(iv) repeat steps (ii)-(iii) *R* times to obtain *R* sets of 2*M* predictive importance scores (which are derived by the random forests method during the fitting process by means of out-of-bag (OOB) error rates)
(v) for each replicate extract the maximum importance score of the permuted features to form the vector $I_{P}^{max}$ of *R* elements
(vi) compare $I_{P}^{max}$ with the *R* importance scores for each of the original features using the Wilcoxon singed-rank test at a statistical significance threshold of 0.05/*M*, to correct for multiple comparisons
(vii) discard all features with *P* values above this threshold and use the unbiased feature subset *S*_*select*_ for further analysis

A detailed layout of the random forests model fitting procedure for the MAL dataset can be found in S1 Text; S1 Script provides the R code to run our feature selection procedure.

### Predictive accuracy

We used two measures to assess and report on the models’ predictive accuracies: (i) the receiver operating characteristic (ROC) curve, which is generated by plotting the true positive rate against the false positive rate (i.e. the observed incidence against the false predicted incidence) at various threshold settings. The area under the curve (AUC) is a measure of predictive accuracy, with an AUC = 1 equating to zero error and an AUC = 0.5 equating to random guessing; and (ii) by means of a confusion matrix, which contrasts the instances of the predicted classes (protected or susceptible) against the observed classes. The misclassification-rates are based on the so-called out-of-bag (OOB) errors [27]. These are derived by iteratively testing the model’s performance against subsets of data left out during the fitting process (recall that each decision tree in a random forests is built on a bootstrapped sample of the original data).

OOB errors represent an estimate of the generalisation error, that is, how well the model would fare against previously unseen data. For the MAL dataset, the OOB error computed on a model fitted only on the selected features would be over-optimistic due to selection bias [34]. Instead, we use our feature selection algorithm inside a five-fold cross-validation loop. Within each fold, the model fitted using the selected features (for each fold the number of selected features may vary) is tested against the left out fold. We report the average AUC across all folds.

## Results

### Analysis of ELISA / Luminex-based immune profile

We first analysed two datasets comprising ELISA and Luminex-derived antibody profiles against *P. falciparum*—specific antigens obtained from prospective cohort studies in Kenya (KEN) and Kenya / Tanzania (KTZ) (see Material and methods). For each dataset, we used a random forests (RF) machine learning approach to predict individual-level protection against clinical immunity over a specified period of time based on (i) measured antibody levels (Ab), (ii) proxies for exposure (such as age or bednet use) (Exp), and (iii) all measured variables (Ab and Exp).

**Fig 1:**
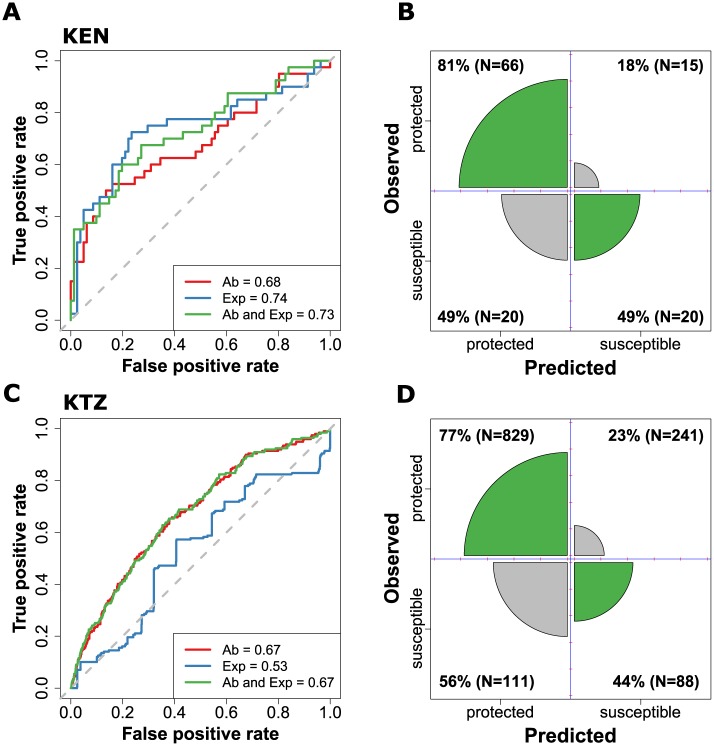
Predictive performance of random forests models of the KEN (top) and KTZ (bottom) datasets. A, C: ROC curves for the random forests models built on antibodies only (red line), exposure proxies only (blue line) and all variables (green line). Area under the curve (AUC) is given in their respective legend, whilst the dashed grey line represents an AUC = 0.5, that is, randomly guessing the individual’s status. B, D: Illustrated confusion matrices derived from models built on all available variables (Ab and Exp). The sizes of the diagonal wedges correspond to the true positive and true negative rates whereas the sizes of the off-diagonal wedges correspond to the false positive and false negative rates.

Fig 1 illustrates the outcome of this analysis by two measures of predictive accuracy: the receiver operator characteristic (ROC) curves (Fig 1A and 1C) and illustrated confusion matrices (Fig 1B and 1D). To our surprise, Ab-levels considered in these studies, including those against previously proposed vaccine targets, such as MSP1 or AMA1, are poor predictors of individual-level protection, with misclassification rates of up to 56%. A weak signal for protective immunity could be found in the KEN dataset using both antibodies and exposure variables. However, a null-model based solely on exposure proxies (age and schizont extract, blue line in Fig 1A) was equally predictive of an individual’s risk of malaria as the more complex model that also included their immune profiles (green line, Fig 1A). High misclassification was also found in the KTZ cohort data (Fig 1C and 1D), which we believe was mainly due to the very young host ages in this study (between 4-18 months at recruitment), where individuals were still experiencing their first malaria infections. Therefore, exposure did not contribute to the model’s predictive performance.

What these results demonstrate is that even in the case where some responses might show univariate statistically significant associations with protection at the population-level, their effect sizes are too small to be able to predict whether someone with elevated titres will be protected during the next transmission season or not. It is equally possible that the data was simply too limited with respect to the age-range and/or the specificity and number of (allelic) antigens considered in the respective assays to identify immune signatures that clearly distinguish clinically immune individuals.

### Analysis of protein microarray data

The protein-microarray-based MAL dataset comprises a much broader set of antigens, representing over 1000 unique proteins, and contained individuals of two different age classes: children between the age of 2-10 years (mean = 5.8, *N* = 149), which were mostly classed as susceptible (117/149), and young adults between 18-25 years (mean = 20.8, *N* = 37), who had predominantly acquired a state of clinical protection (33/37 individuals remained symptom-free).

**Fig 2:**
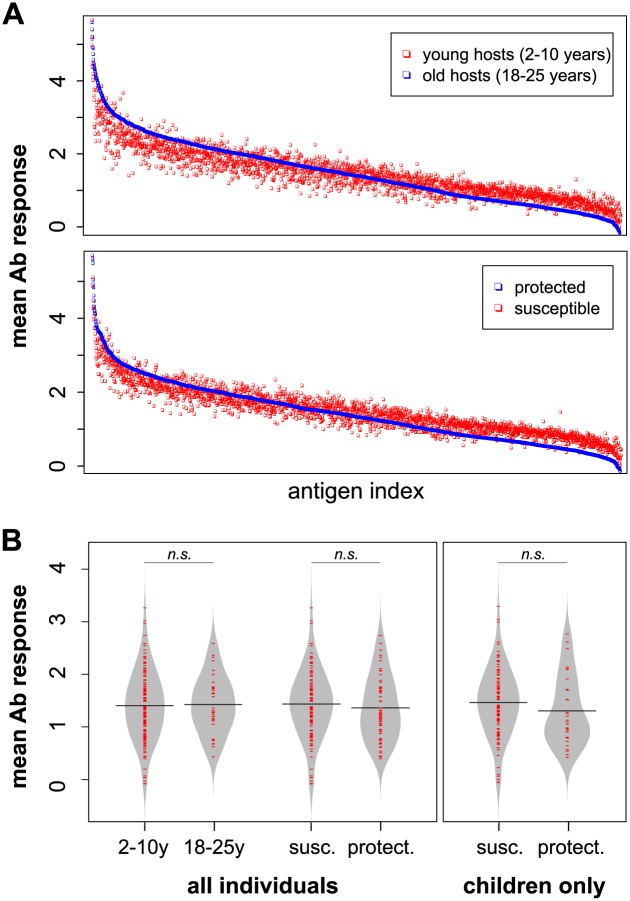
Population-level mean antibody (Ab) responses for the protein microarray data. A: Mean antibody responses stratified by age (2-10y vs. 18-25y, top) or immune status (protected vs. susceptible, bottom) and sorted by decreasing intensity of one of the strata (old individuals, top, and protected individuals, bottom); each dot represents the mean response to an individual antigen. There is a noticeable shift in the immune profiles of the older and/or protected individuals, with high intensity responses being further elevated and low intensity responses being reduced. B: Beanplots showing the mean and distribution of the immune responses stratified by age class (children and young adults) or immune status for all individuals, or stratified by immune status considering children only. There was no statistically significant difference between the respective means (*P* > 0.2 in all cases; Welch’s two-sample *t*-test).

Fig 2A shows the mean antibody measures against all antigens stratified by either age or infection outcome. In both cases we find a consistent, qualitative shift in the immune profiles of protected and older individuals where sets of high-titre antibodies are further elevated and sets of low-titre responses further reduced. Importantly, and as shown in the beanplots [35] in Fig 2B, there is no notable difference in the mean reactivity between the various strata (in all three cases *P* > 0.2, Welch two sample *t*-test), implying that clinical protection within this dataset is not characterised by an increase in overall reactivity.

### Predictive immune responses develop late in childhood

**Fig 3:**
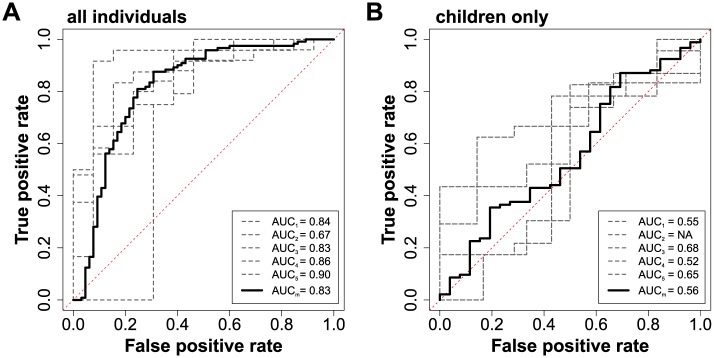
Immune signatures found in protein microarray data are predictive of clinical immunity but only when considering all individuals. ROC curves for each cross-validation fold (dashed black line) and the mean ROC curve (solid black line) for random forests models fitted to A: all individuals (2-25 years) B: children only (2-10 years) (the dashed red line represents an AUC = 0.5, that is, randomly guessing the individual’s status).

To elucidate the most predictive antigens at the individual-level in this dataset, where the number of immune responses (*M* = 2320) was much larger than the number of samples (*N* = 186), we performed feature selection inside a five-fold cross-validation loop (see Material & methods and S1 Text). We first fitted a model to all individuals (2-25 years) using all available predictors (2320 antigenic responses, age and parasite status at screening). The average AUC across all testing folds was 0.83 (Fig 3A), exhibiting very good discrimination between the protected and susceptible individuals. However, this predictive performance dropped considerably when the model was identified using only the children (2-10 years), average AUC across four of the five folds was 0.56 (Fig 3B). For one of the cross-validation folds, no predictors were retained by the feature selection algorithm, so no model could be built.

**Fig 4:**
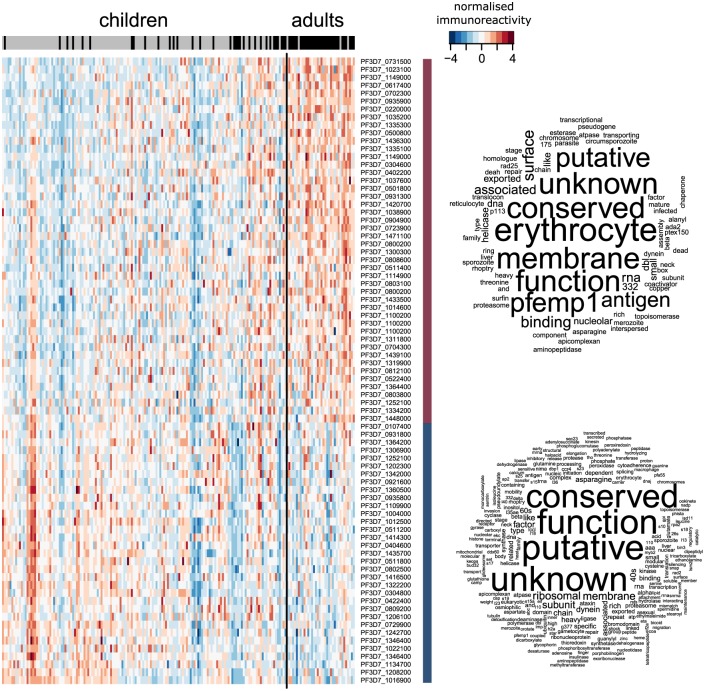
Immunoreactivity heatmap of feature-selected antigens from the MAL dataset when considering all individuals. Antigens identified as important predictors for clinical immunity show either an increase or decrease in intensity as individuals get older under repeated exposure to the parasite. The columns are the responses from each individual, ordered by age (young to old, from left to right), and separated into children (≤ 10 years) and adults (≥ 18 years). The bar at the top indicate the individual’s protective status (grey: susceptible, black: protected), highlighting the strong correlation between age and clinical protection. The word cloud of the protein product description stratified by whether the response had an increasing or decreasing trend with age, is based on the selected features and any other proteins they are highly correlated with (*ρ* > 0.8).

Fig 4 shows a heatmap of immunoreactivity using the set of antigens selected in at least one of the cross-validation folds, and a word cloud for the protein product description based on the selected features and any other proteins they are highly correlated with (*ρ* > 0.8). The set of predictive antigens consists of those with either an increasing or a decreasing response as individuals grow older and gain clinical protection. Interestingly, none of the 47 proteins which showed an increasing response with age were correlated with others. Whereas, 232 proteins were correlated with the 32 responses that exhibited a decreasing response with age. This temporal pattern, as well as the strong relationship between age and immune status, suggests that the change in the immune responses that allows us to distinguish susceptible from clinically immune individuals (at least as measured by the protein microarray) is taking shape through continuous exposure to the malaria parasite during childhood, but does not fully develop until early adulthood. In part, this explains why we see such an extensive degradation to model performance when only the children are considered, and why 79 antigens were selected for the model with all individuals, but only 9 when considering only the children. Moreover, whilst age was an important predictor for all individuals (coming up in all five-folds), it was never selected for the model based on only the children. The word cloud suggests that the responses that increase with age are related to surface variable antigens, whilst the ones that decrease consist of a number of conserved proteins of (currently) unknown function. S4 Data contains the complete list of selected antigens and their annotations.

## Discussion

Despite decades of intensive research, we still do not know which antigens are central in the induction of immune responses that protect against clinical malaria. Small effect sizes of individual responses and the often polymorphic nature of many immune targets, coupled with considerable inter-individual heterogeneity, are partially to blame for the lack of a clear relationship between a measured response and the level of protection against malaria it offers. Osier and colleagues [3, 12] have previously proposed that protection could in fact be due to the accumulation of responses against sets and or specific combinations of antigens in a threshold-dependent manner, which would certainly help to overcome the issue of individual responses having small effect sizes. However, the number of combinations and possible interactions between antigens that need to be tested and compared across studies to draw robust conclusions can easily become infeasible using standard statistical approaches. Mainly due to identifiability issues, where insufficient samples are available to estimate each model parameter. The predictive modelling framework based on machine learning described here offers a systematic approach to consider all possible combinations of measured antibodies and to extract the most distinguishing features from these high-dimensional datasets in a hypothesis-free way. Furthermore, as the outcome of this approach is the predictive accuracy, i.e. the degree by which the model can predict the response at the level of the individual, results are easily comparable across studies, in contrast to *P*-values, which are strongly dependent both on sample size and the chosen statistical test and/or model.

Our analysis of different sets of cohort data based on immune profiles against relatively limited sets of *P. falciparum*-specific antigens demonstrated that commonly assumed immune correlates and potential vaccine candidates (e.g. MSP-1, MSP-2, or AMA-1) are poor predictors of clinical protection in children. Apart from small effect sizes and the fact that in most studies only a limited set of target alleles are investigated, the age-range of individuals considered in these studies may also play a role in explaining our findings. That is, previous studies have shown that protection against life-threatening disease might be acquired early in life after only a few infections (see e.g. [36]). On the other hand, clinical protection is a more gradual process whereby the probability of a clinical episode declines slowly under repeated exposure. This may make the identification of immune signatures that are highly predictive at the individual-level problematic, unless the considered age ranges, and therefore the levels of cumulative exposure, are sufficiently broad.

What our results also point towards is that a diverse set of antigens must be considered to robustly identify predictive immune signatures. The distinct pattern that we found in the data based on protein-microarrays was characterised by an exposure-driven change in the responses to several surface-expressed and internal, conserved and polymorphic parasite proteins. Importantly, a small subset of antigens was sufficient to predict an individual’s risk of presenting with a symptomatic malaria infection during the following transmission season with a high degree of accuracy, at least within a set of individuals covering a suitably wide age-range. In contrast to previous studies, the difference in the immune profiles between the two phenotypes consisted of both increased and decreased responses to certain antigens, which intensified with age. Whereas those responses which showed increasing intensities with exposure contained various known immune targets, such as PfEMP1, the responses that decreased with age were mostly against proteins of unknown function. It is possible that these decreasing responses are an artefact of the microarray data as they mostly consisted of low-titre responses (see S1 Text), i.e. those with small signal-to-noise ratios. In their original analysis, Crompton *et al*. [8] only considered responses immunogenic if they were 2 SDs above the controls, whereas we considered all responses and are therefore more likely to pick up immunologically irrelevant features. Further investigations are therefore required to verify if and to what degree these responses relate to (protective) immunological pathways.

The fact that the predictive responses showed such a strong association with age also begs the question whether they are true targets of the protective responses or whether they simply mirror infection histories; the latter is probably more plausible for the internal rather than surface-expressed antigens. Finding reliable markers of previous/cumulative exposure is arguably one of the most fundamental problems in correctly identifying antibody-based correlates of protection. Not only are they important in assessing how many undetected infections get past passive and/or active surveillance during follow-up periods, but the ability to discern between cases where someone is truly protected, i.e. infected but without showing clinical symptoms, or simply has a lower exposure risk, would allow us to define reliable phenotypes. A more direct approach would be the inclusion of only those individuals with documented infections [37]. However, this relies on a much higher sampling frequency or reliable markers of recent infections. Age, homestead location and previous malaria incidence are common markers, but none of these directly quantify how often the immune system was challenged by a *P. falciparum* infection. Using a predictive framework, Helb and colleagues [21] recently established an alternative way for estimating recent exposure by identifying key antigens that were most predictive of days since the last *P. falciparum* infection and incidence of symptomatic malaria during the previous year. The immune signature identified in our analysis is not so much indicative of recent infections but more of repeated challenges over years of continuous exposure.

In order to make our findings and methodological approach relevant, not only for understanding the process of natural acquired immunity to malaria, but also with regards to future intervention measures, including vaccines, our results need to be validated against independent datasets. In the first instance these should involve replicate studies in similar transmission settings, including the follow up of individuals over successive transmission seasons to test for the robustness of the identified immune signatures. More importantly, though, is the issue of differentiating between individuals who are protected and those who did not get challenged. In the studies considered here this was not a major concern as they were all based in moderate to high transmission settings with most children experiencing a clinical episode over the study periods. However, in areas of lower transmission intensities this is a pressing concern. One obvious way around this would be the use of longitudinal cohort studies with active surveillance. Another, and much cheaper option would be to exploit data analytical approaches as employed by Helb and colleagues [21].

A good correlate of protection should be a universal biomarker reflecting an immune response that prevents the parasite from causing clinical and life-threatening disease. Our results need to be validated against independent datasets to investigate whether the predictive signatures we identified perform equally well in different malaria endemic settings and to test to what degree they truly capture functional immune mechanisms. In that respect we believe that predictive models offer clear advantages over univariate association analyses. Not only is predictive accuracy directly comparable between studies, but these frameworks also provide a systematic way to consider all putative correlates of protection whilst reducing the chances of false discoveries. With the advent of more detailed and complex big data sets in the field of malaria immuno-epidemiology these models should therefore be considered more prominently alongside standard statistical approaches in an attempt to unravel the complex interplay between exposure and infection outcome in *P. falciparum* malaria.

## Supporting information

S1 Data KEN dataset. (CSV)

S2 Data KTZ dataset. (CSV)

S3 Data MAL dataset. (CSV)

S4 Data Table of selected antigens identified as important predictors for clinical immunity for all individuals and for children only. (CSV)

S1 Text Predictive modelling of protein microarray data. This document provides a detailed explanation of the predictive modelling approach used to analyse the protein microarray data of [8]. The protein microarray contained ∼ 23% of the *P. falciparum* 5,400 protein proteome. It was used to test plasma reactivity from 186 individuals between the ages of 2-10 years and 18-25 years in Mali before the 6-month malaria season. Here we investigated whether the imune profiles were predictive of protection from clinical episodes using a random forests machine learning approach, preceeded by a modified mProbes [32]/xRF [33] feature selection.
(PDF)

S1 Script R script file for running the feature selection procedure. (R)
