# Supplementary material for: Identification of immune signatures predictive of clinical protection from malaria
Source: PLoS Comput Biol. 2017 Oct 24;13(10):e1005812. doi: 10.1371/journal.pcbi.1005812 (PMC5669498; doi:10.1371/journal.pcbi.1005812)
Supplement: S1 Text — This document provides a detailed explanation of the predictive modelling approach used to analyse the protein microarray data of [8]. The protein microarray contained ∼ 23% of the P. falciparum 5,400 protein proteome. It was used to test plasma reactivity from 186 individuals between the ages of 2-10 years and 18-25 years in Mali before the 6-month malaria season. Here we investigated whether the imune profiles were predictive of protection from clinical episodes using a random forests machine learning approach, preceeded by a modified mProbes [32]/xRF [33] feature selection. (PDF) [file pcbi.1005812.s005.pdf]

Predictive modelling of protein microarray data

Identification of immune signatures predictive of clinical protection from malaria

John Joseph Valletta and Mario Recker

28 September 2017

This document provides a detailed explanation of the predictive modelling approach used to analyse the protein microarray data of Crompton et al. (2010). The protein microarray contained $\sim 23\%$ of the *P. falciparum* 5,400 protein proteome. It was used to test plasma reactivity from 186 individuals between the ages of 2-10 years and 18-25 years in Mali before the 6-month malaria season. Here we investigated whether the imune profiles were predictive of protection from clinical episodes using a random forests machine learning approach, preceeded by a modified mProbes (Huynh-Thu et al. (2012))/xRF (Nguyen, Huang, and Thuy (2014)) feature selection.

Load the data and required libraries

```
rm(list = setdiff(ls(), lsf.str())) # clear workspace

# Libraries
library(caret) # findCorrelation(...)
library(RColorBrewer) # pretty colours
library(gplots) # heatmap.2(...)
library(scales) # alpha
source("feature_selection.R") # functions for feature selection/predictive modelling

# Constants
DATA_PATH <- file.path("..", "Data", "AClean", "MAL.csv")
NTREE <- 20001 # number of trees in forest
NREPEAT <- 7*15 # number of repeats for mProbes/xRF
NSEED <- 1983 # for reproducibility
```

```
# Load data
df <- read.csv(DATA_PATH, header=T, check.names=FALSE)
df$ParasiteAtScreen <- as.numeric(df$ParasiteAtScreen) # amenable for findCorrelation()
head(df)[, c(1, 2, 3, 6, 2327, 7:9)] # show meta data and reactivity of some proteins
```

```
##   SubjectID Age Gender ParasiteAtScreen      Status MAL13P1.234-e1s2
## 1         1   4 female                2   protected        1.6080768
## 2         2   4 female                1 susceptible        0.1242947
## 3         3   4 female                1 susceptible       -0.1815251
## 4         4   4   male                2 susceptible        1.7366575
## 5         5   4 female                1   protected        0.8531175
## 6         6   4 female                1 susceptible        0.0148640
##   PF13_0320-e13s2 PF11_0479-e3s1
## 1       1.6343895      2.1518086
## 2       0.9227035      0.3648743
## 3       0.5041609      0.3773841
## 4       3.6187181      3.7815985
## 5       1.3092684      1.2885002
## 6       1.6237722      1.7407992
```

We now define our feature space, `X`, to contain the normalised microarray data (2320 columns) plus two proxy exposure variables, `Age` and `ParasiteAtScreen` from 186 individuals. We also define our response variable, `Y`, as the reported incidence of a symptomatic malaria infection during the follow-up period, where individuals are classified either as `susceptible` (one or more recorded symptomatic infections during follow-up) or `protected` (no recorded symptomatic infections).

```
ignore <- c("SubjectID", "Gender", "NEpisodes", "TimeToFirstEpisode", "Status")

X <- df[, !(colnames(df) %in% ignore)] # all Ab-levels, Age and Parasit status at screening
Y <- df$Status
dim(X)
```

```
## [1]  186 2322
```

```
length(Y)
```

```
## [1] 186
```

Temporary remove strongly linearly correlated features

To avoid biasing the variable importance measures computed by the random forests (Toloşi and Lengauer (2011)), highly correlated responses (above a Pearson correlation coefficient of $\rho = 0.8$) were temporary excluded from the analysis. Note that these correlated variables are reintroduced in the interpretation stage if any of the features they are associated with are included in the final model.

```
rho = cor(X, method="pearson")
iiRemove <- findCorrelation(rho, cutoff=0.8) # indices to remove to reduce pair-wise corr
X <- X[, -iiRemove]
dim(X)
```

```
## [1]  186 1482
```

Feature selection and predictive modelling

The remaining covariates undergo a rigorous supervised feature selection process, based on the mProbes (Huynh-Thu et al. (2012)) and xRF (Nguyen, Huang, and Thuy (2014)) as follows:

1. fit a large random forests considering all features; keep only the top 30% ranked features according to their variable importance measure for the subsequent steps (this step is justified because most importance scores are very low and it is therefore highly unlikely that any of these features will have sufficient predictive capacity)
2. permute the values of every predictor (i.e. antibody response), X_i ($i = 1 \ldots M$, where M is the total number of predictors), and add these to the original feature space, S_X, to generate an extended feature space $S_{X,P}$ (P represent the permuted features), which now has the dimension $N \times 2M$ (where N is the total number of individuals)
3. build a random forests model from $\{S_{X,P},\ Y\}$, where Y is the response variable (i.e. protected/susceptible)
4. repeat steps (2)-(3) R times to obtain R sets of $2M$ predictive importance scores (which are derived by the random forests method during the fitting process by means of out-of-bag (OOB) error rates)
5. for each replicate extract the maximum importance score of the permuted features to form the vector I_P^{max} of R elements

6. compare I_P^{max} with the R importance scores for each of the original features using the Wilcoxon singed-rank test at a statistical significance threshold of $0.05/M$, to correct for multiple comparisons
7. discard all features with P values above this threshold and use the unbiased feature subset S_{select} for further analysis

```
set.seed(NSEED) # for reproducible cross-validation folds
out <- pred_modelling_pipeline(X, Y, kFold=5, rKeep=0.3, nRepeat=NREPEAT,
                               ntree=NTREE, description="Mali") # results are stored on disk
```

```
## Fold 1/5
## a) Fitting random forest to training data (all predictors)...
## b) Running mProbes feature selection algorithm on the kept predictors...
## c) Compute statistical significance between real and random features...
## d) Fit final model to top predictors...
## Fold 2/5
## a) Fitting random forest to training data (all predictors)...
## b) Running mProbes feature selection algorithm on the kept predictors...
## c) Compute statistical significance between real and random features...
## d) Fit final model to top predictors...
## Fold 3/5
## a) Fitting random forest to training data (all predictors)...
## b) Running mProbes feature selection algorithm on the kept predictors...
## c) Compute statistical significance between real and random features...
## d) Fit final model to top predictors...
## Fold 4/5
## a) Fitting random forest to training data (all predictors)...
## b) Running mProbes feature selection algorithm on the kept predictors...
## c) Compute statistical significance between real and random features...
## d) Fit final model to top predictors...
## Fold 5/5
## a) Fitting random forest to training data (all predictors)...
## b) Running mProbes feature selection algorithm on the kept predictors...
## c) Compute statistical significance between real and random features...
## d) Fit final model to top predictors...
```

Note: The implementation of the predictive modelling pipeline is parallelised. However, with the current version we cannot set the same random seed for every core used, hence results will vary slightly if run again. The cross-validation folds are the same though, so the differences are small.

Model performance

Out-of-bag (OOB) errors represent an estimate of the generalisation error, that is, how well the model would fare against previously unseen data. For the MAL dataset, the OOB error computed on a model fitted only on the selected features would be over-optimistic due to selection bias (Ambroise and McLachlan (2002)). Instead, we used our feature selection algorithm inside a five-fold cross-validation loop. Within each fold, the model fitted using the selected features (for each fold the number of selected features may vary) is tested against the left out fold. We report ROC (receiver operating characteristic) curves and auc (area under the curve) for all folds and their average.

```
# Load results data
auc <- readRDS(file.path(getwd(), "Mali", "auc.rds"))
ROC <- readRDS(file.path(getwd(), "Mali", "ROC.rds"))

# Plot results
```

```
par(pty="s", cex.axis=1.5)

# Plot first fold ROC curve
plot(ROC[["Fold1"]], lty=2, font.lab=2, cex.lab=2, col="grey", lwd=2)
legStr <- paste("AUC1 =", format(auc[["Fold1"]], digits=2)) # legend string

# Plot rest of the folds
for (k in 2:5)
{
    fold <- paste0("Fold", k)
    plot(ROC[[fold]], lty=2, font.lab=2, cex.lab=2, col="grey", lwd=2, add=T)
    legStr <- c(legStr, paste0("AUC", k, " = ", format(auc[[fold]], digits=2)))

}
plot(ROC[["Average"]], lty=1, lwd=4, font.lab=2, cex.lab=2, add=T)
legStr <- c(legStr, paste("AUC avg", "=", format(auc[["Average"]], digits=2)))
abline(a=0, b=1, col="red", lty=2, lwd=2)
legend("bottomright", legStr, col="black", lty=c(rep(2, 5), 1), cex=1.2)
```

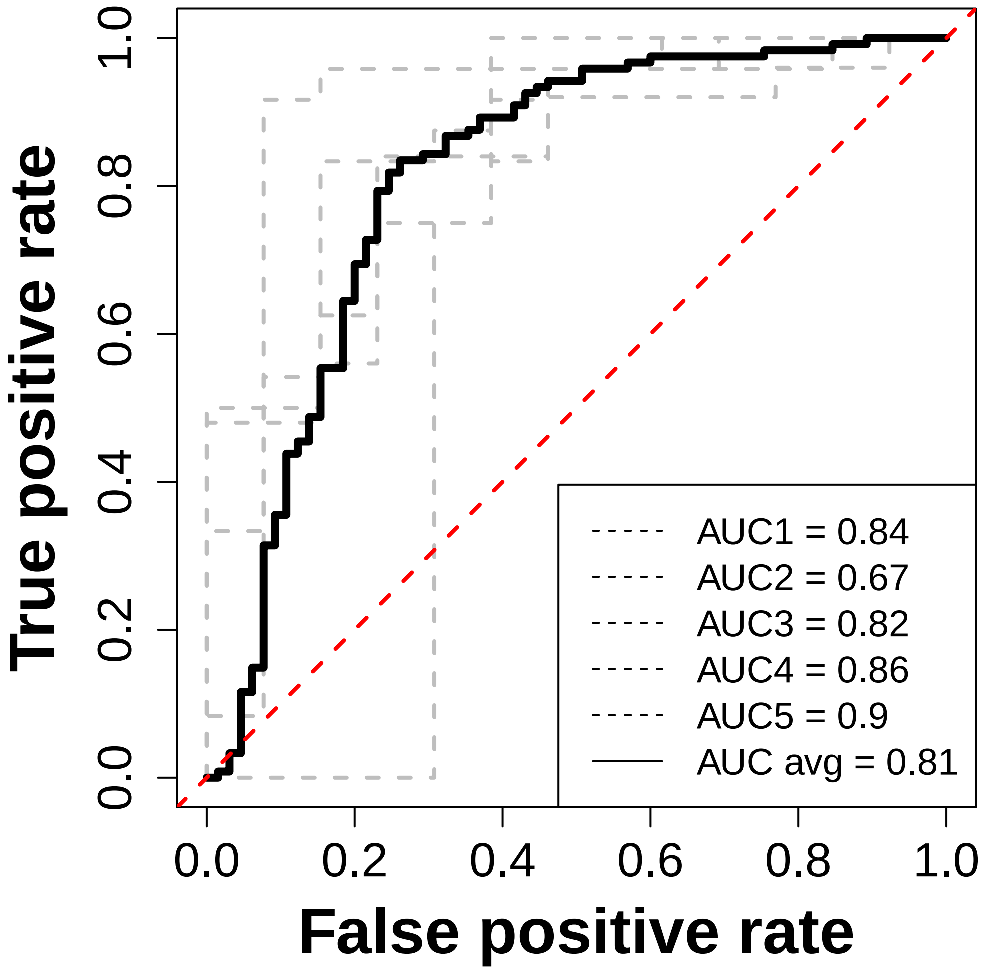

Mean antibody response of top predictors

Let us plot the mean reactivity across all samples for all proteins and shade the ones that go up or down with age.

```
# Read list of top proteins
top <- as.character(unlist(read.csv((file.path(getwd(), "Mali", "topPredictors.csv")))))
top <- unique(top[!(is.na(top)) & !(top %in% c("Age", "ParaisteAtScreen"))])

# Find if the general trend is up/down with age
beta0 <- c()
betaAge <- c()
for (p in top) # p = protein
{
    # Find if it's going up/down with age
    myForm <- as.formula(paste0("`", p, "` ~ Age"))
    fit <- lm(myForm, data=df) # a crude fit
    beta0 <-c(beta0, coef(fit)[["(Intercept)"]])
    betaAge <- c(betaAge, coef(fit)[["Age"]])
}
```

```
# Prepare protein IDs
ignore <- c("SubjectID", "Gender", "NEpisodes", "TimeToFirstEpisode", "Status", "Age", "ParasiteAtScree
abCol <- setdiff(colnames(df), ignore) # antibody level columns
upID <- top[betaAge>0] # IDs that go up with age
dnID <- top[betaAge<0] # IDs that go down with age
upCol <- "#e41a1c" # red
dnCol <- "#377eb8" # blue
noCol <- "lightgrey"

# Compute mean across all samples
meanAb <- apply(df[, match(abCol, colnames(df))], 2, mean)
names(meanAb) <- abCol # antibody columns
meanAb <- sort(meanAb, decreasing=T)
thisCol <- ifelse(names(meanAb) %in% upID, upCol, ifelse(names(meanAb) %in% dnID, dnCol, noCol))

# Plot mean reactivity across all sample
ii <- which(thisCol %in% noCol) # list of indices to plot
plot(ii, meanAb[ii], col=thisCol[ii], pch=20, xlab="antigen index", ylab="mean Ab response")
ii <- which(thisCol %in% c(upCol, dnCol))
points(ii, meanAb[ii], bg=thisCol[ii], pch=21)
legend("topright", c("Up with age", "Down with age", "Unselected proteins"),
       fill=c(upCol, dnCol, noCol))
```

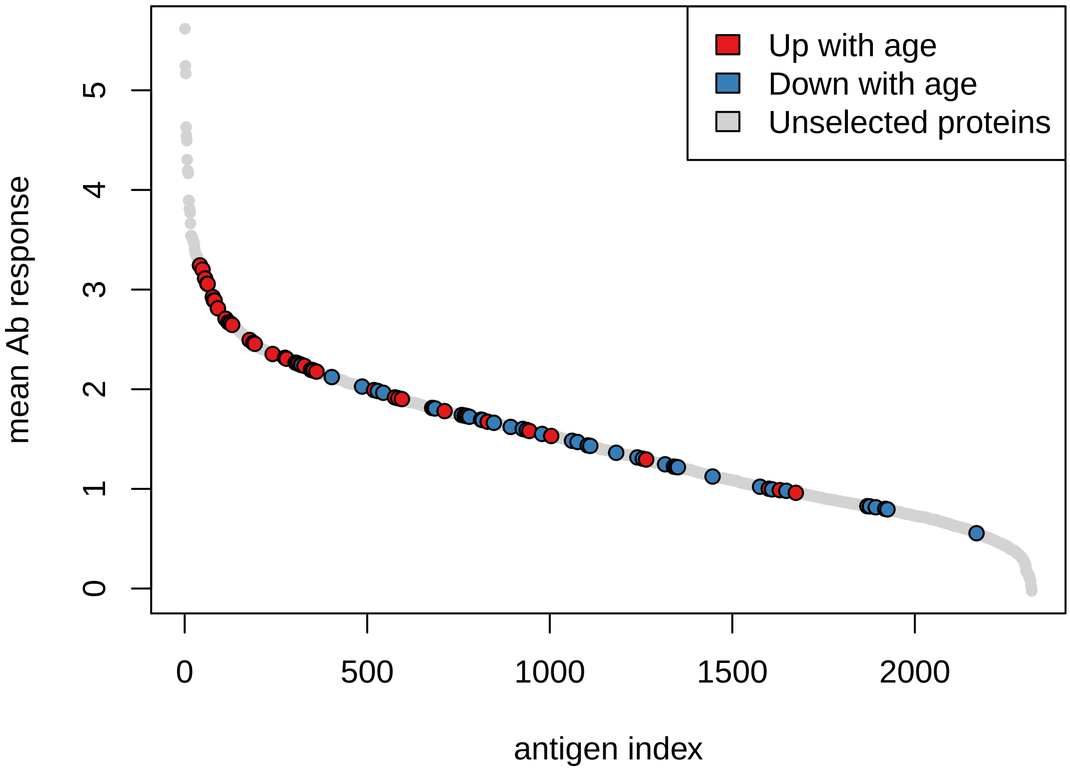

Visualise predictive patterns

To elucidate on the pattern that the model found to be predictive of clinical protection we plot the individual responses as a heatmap. Here we order them by age to see how this pattern emerges as individuals get older.

Note: Random forests mine for predictive patterns made up of any form of (possibly non-linear) combinations across different proteins. Thus, heatmaps on their own may not always reveal a clear picture of the predictive pattern found. Partial dependency plots which show the relationship between an outcome and one or two features, whilst marginalising all others, are also useful complementary visualisation tools.

```
# Get data set ordered by age
df <- df[order(df$Age), ]
columnColour <- ifelse(df$Status=="protected", "black", "grey")
dataMatrix <- t(as.matrix(df[, match(top, colnames(df))]))

# Plot heatmap
heatmap.2(dataMatrix,
          # dendrogram control
          distfun=function(x) dist(x, method="euclidean"), # canberra, mikowski
          hclustfun=function(x) hclust(x, method="ward.D2"),
          dendrogram="none",
          scale="row",
          Rowv=TRUE,
          Colv=FALSE,
          # colour map
          col=rev(brewer.pal(11,"RdBu")),
          # level trace
          trace="none", # Remove ugly trace lines
```

```
          main="", # title
          # color key + density info
          key=TRUE, # Display key or not
          keysize=0.9, # Size of colour key
          density.info="none", # Histogram or None
          # Row/Column Labeling
          labRow=top,
          labCol="",
          ColSideColors=columnColour,
          cexRow=0.5,
          colsep=min(which(df$Age > 10)),
          sepcolor="black",
          sepwidth=c(0.25, 0.25),
          srtCol=90)
legend("topright", legend=c("Protected", "Susceptible"), fill=c("black", "grey"), cex=0.7)
```

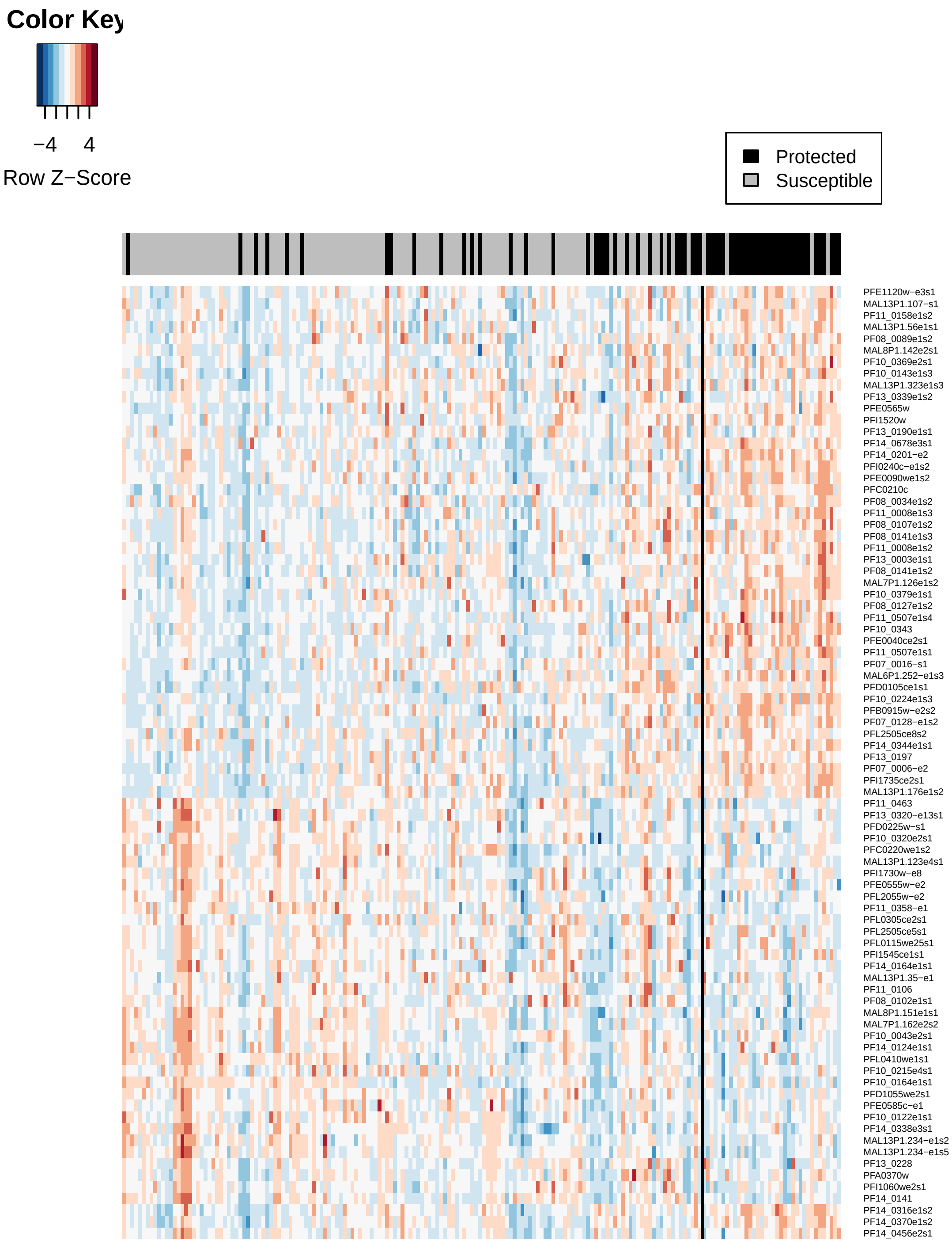

Recall that initially we temporary removed strongly linearly correlated features. To help with interpreting these results we bring back any responses which were correlated to any of the selected features.

```
ID <- c() # complete list of selected protein IDs
for (protein in top) # p = protein
{
    corVars <- rownames(rho)[abs(rho[, match(protein, colnames(rho))])>0.8] # correlated vars
    ID <- union(ID, corVars)
}
```

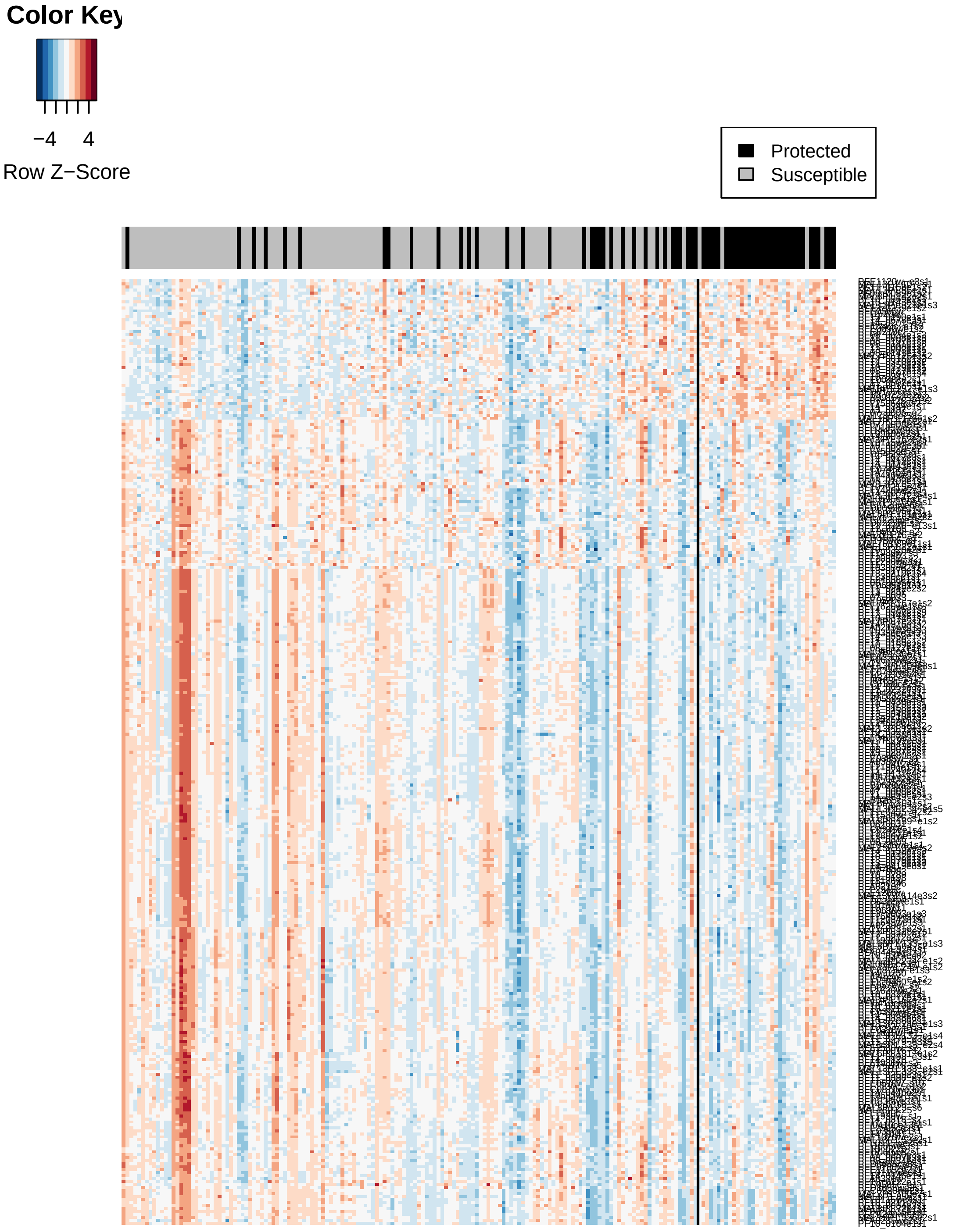

The heatmap including all responses isn't useful because by definition we are plotting strongly correlated responses. Instead the full list of protein IDs can be queried on PlasmoDB or similar databases to investigate whether some of these responses cluster together by biological function. In the manuscript we did this by producing a word cloud for proteins that go up or down with age.

```r
sessionInfo()
```

```
## R version 3.4.1 (2017-06-30)
## Platform: x86_64-apple-darwin15.6.0 (64-bit)
## Running under: macOS Sierra 10.12.6
##
## Matrix products: default
## BLAS: /Library/Frameworks/R.framework/Versions/3.4/Resources/lib/libRblas.0.dylib
## LAPACK: /Library/Frameworks/R.framework/Versions/3.4/Resources/lib/libRlapack.dylib
##
## locale:
## [1] en_GB.UTF-8/en_GB.UTF-8/en_GB.UTF-8/C/en_GB.UTF-8/en_GB.UTF-8
##
## attached base packages:
## [1] parallel  stats     graphics  grDevices utils     datasets  methods
## [8] base
##
## other attached packages:
##  [1] class_7.3-14        ROCR_1.0-7          doParallel_1.0.10
##  [4] iterators_1.0.8     foreach_1.4.3       randomForest_4.6-12
##  [7] scales_0.5.0        gplots_3.0.1        RColorBrewer_1.1-2
## [10] caret_6.0-77        ggplot2_2.2.1       lattice_0.20-35
##
## loaded via a namespace (and not attached):
##  [1] Rcpp_0.12.12       lubridate_1.6.0    gtools_3.5.0
##  [4] assertthat_0.2.0   rprojroot_1.2      digest_0.6.12
##  [7] ipred_0.9-6        R6_2.2.2           plyr_1.8.4
## [10] backports_1.1.0    stats4_3.4.1       evaluate_0.10.1
## [13] rlang_0.1.2        lazyeval_0.2.0     gdata_2.18.0
## [16] kernlab_0.9-25     rpart_4.1-11       Matrix_1.2-11
## [19] rmarkdown_1.6      splines_3.4.1      CVST_0.2-1
## [22] ddalpha_1.2.1      gower_0.1.2        stringr_1.2.0
## [25] munsell_0.4.3      compiler_3.4.1     pkgconfig_2.0.1
## [28] dimRed_0.1.0       htmltools_0.3.6    nnet_7.3-12
## [31] tibble_1.3.4       prodlim_1.6.1      DRR_0.0.2
## [34] codetools_0.2-15   RcppRoll_0.2.2     dplyr_0.7.3
## [37] withr_2.0.0        MASS_7.3-47        bitops_1.0-6
## [40] recipes_0.1.0      ModelMetrics_1.1.0 grid_3.4.1
## [43] nlme_3.1-131       gtable_0.2.0       magrittr_1.5
## [46] KernSmooth_2.23-15 stringi_1.1.5      reshape2_1.4.2
## [49] bindrcpp_0.2       timeDate_3012.100  robustbase_0.92-7
## [52] lava_1.5           tools_3.4.1        glue_1.1.1
## [55] DEoptimR_1.0-8     purrr_0.2.3        survival_2.41-3
## [58] yaml_2.1.14        colorspace_1.3-2   caTools_1.17.1
## [61] knitr_1.17         bindr_0.1
```

References

Ambroise, Christophe, and Geoffrey J McLachlan. 2002. "Selection bias in gene extraction on the basis of microarray gene-expression data." *Proceedings of the National Academy of Sciences of the United States of America* 99 (10): 6562–6. doi:10.1073/pnas.102102699.

Crompton, Peter D, Matthew A Kayala, Boubacar Traore, Kassoum Kayentao, Aissata Ongoiba, Greta E Weiss, Douglas M Molina, et al. 2010. "A prospective analysis of the Ab response to Plasmodium falciparum before and after a malaria season by protein microarray." *Proceedings of the National Academy of Sciences of the United States of America* 107 (15): 6958–63.

Huynh-Thu, V. A., Y. Saeys, L. Wehenkel, and P. Geurts. 2012. "Statistical interpretation of machine learning-based feature importance scores for biomarker discovery." *Bioinformatics* 28 (13): 1766–74. doi:10.1093/bioinformatics/bts238.

Nguyen, TT, JZ Huang, and TN Thuy. 2014. "Unbiased Feature Selection in Learning Random Forests for High Dimensional Data." *The Scientific World Journal* 2015: 1–18. http://downloads.hindawi.com/journals/tswj/aip/471371.pdf.

Toloşi, Laura, and Thomas Lengauer. 2011. "Classification with correlated features: Unreliability of feature ranking and solutions." *Bioinformatics* 27 (14): 1986–94. doi:10.1093/bioinformatics/btr300.
